# Overcoming Challenges and Opening New Opportunities in Glycoproteomics

**DOI:** 10.3390/biom3020270

**Published:** 2013-03-22

**Authors:** Ten-Yang Yen, Sucharita M. Dutta, Christina Litsakos-Cheung, Alejandro A. Corona, Leslie C. Timpe, Bruce A. Macher

**Affiliations:** 1 Department of Chemistry and Biochemistry, San Francisco State University, San Francisco, CA 94132, USA; E-Mails: ryen@sfsu.edu (T.-Y.Y.); cmlitsakos@gmail.com (C.L.C.); aacorona@mail.sfsu.edu (A.A.C.); lestimpe@sbcglobal.net (L.C.T.); 2 Thermo Fisher Scientific, San Jose, CA 95134, USA; E-Mail: sucha227@gmail.com

**Keywords:** glycoproteomics, *N*-linked glycosites, hydrazide-modified magnetic beads, Orbitrap, tandem mass spectrometry, ^18^O labeling, deamidation

## Abstract

Glycoproteomics has emerged as a prime area of interest within the field of proteomics because glycoproteins have been shown to function as biomarkers for disease and as promising therapeutic targets. A significant challenge in the study of glycoproteins is the fact that they are expressed in relatively low abundance in cells. In response, various enrichment methods have been developed to improve the detection of glycoproteins. One such method involves their capture via oxidation of their glycan chains and covalent attachment with hydrazide resins which, when catalyzed by PNGase F, release *N*-linked glycans and convert the glycosite Asn to Asp; this conversion is identifiable with LC/ESI-MS/MS as a corresponding increase of 0.984 Da in molecular weight. The present study builds on this body of work, providing evidence of three additional strategies that improve glycoprotein identification: (1) use of a high resolution mass spectrometer—the Q Exactive MS—which delivers 2–3 times more glycoprotein identifications than a low resolution MS; (2) optimization of instrument settings and database search parameters to reduce misidentification of *N*-linked glycopeptides to ~1 percent; and (3) labeling glycopeptides with ^18^O during PNGase F treatment to locate *N*-linked glycosites within peptides containing multiple *N*-linked sequons.

## 1. Introduction

The development of modern mass spectrometers and software to link mass spectra to sequences in protein databases has provided opportunities to obtain systematic information on proteins and their posttranslational modifications [1,2]. Initial proteomic studies focused on the development of methods to enrich for subcomponents of the proteome and on the advancement of quantitative analytic approaches [3]. Although substantial progress has been made, significant challenges remain.

Early proteomic surveys of the major proteins expressed in various cell types identified few glycoproteins, consistent with their relatively low abundance in most cell types [4]. Given their critical biological functions and their usefulness as biomarkers for various diseases, significant efforts to identify glycoproteins initially focused on their detection in readily available body fluids [5,6,7,8]. With the development of methods for removing the major plasma proteins, a large number of glycoproteins were identified in plasma [9].

Our laboratory is focused on the characterization of cell surface and secreted glycoproteins expressed by normal human breast epithelia and various subtypes of breast cancer cells. Our current research aims to identify alterations associated with oncogenesis in this subproteome as well as to identify biomarker candidates for various breast cancer subtypes. In the course of this research, we have developed an efficient approach to enrich for the subproteome. Despite considerable progress in this domain, a pressing need remains for more comprehensive coverage of the glycoproteome. The availability of new mass spectrometers with advanced design features offers a promising avenue for addressing existing gaps. We report here on the results of a study we conducted to determine the extent to which such instrumentation may increase the depth of coverage of the cell surface and secreted glycoproteome.

Glycoproteomic identifications have been made based on the detection of *N*-linked glycopeptides, non-glycosylated tryptic peptides or a combination of both and the advantages of using one or both have been presented (see ref. 10 for example) [10]. Identification of glycoproteins based on *N*-linked glycopeptides generally depends on detection of the deglycosylated peptide derived from PNGaseF treatment. PNGaseF is an amidase that cleaves between the innermost *N*-linked core GlcNAc and the Asn residue to which it is covalently linked. PNGaseF catalyzes the conversion of the *N*-linked Asn to an Asp residue, with a resulting increase of 0.984 Da in molecular weight [11] Chemical deamidation that can occur during sample preparation [12,13,14,15] produces the same mass shift as the PNGaseF-catalyzed reaction, leading to potential misidentification of *N*-linked glycosylation sites. To reduce such misidentification, inclusion of O^18^ in the PNGaseF reaction—resulting in the incorporation of O^18^ rather than of O^16^ during hydrolysis—has been found effective [16]. In the current study, we revisit the issue of chemical induced deamidation *vs.* PNGaseF mediated conversion of Asn to Asp residues. Specifically, we use O^18^ labeling in the PNGaseF mediated glycosite identification to determine how often misidentification of *N*-linked sites due to chemical deamidation occurs, especially within glycopeptides containing more than one *N*-linked consensus sequence. 

## 2. Results and Discussion

**Figure 1:**
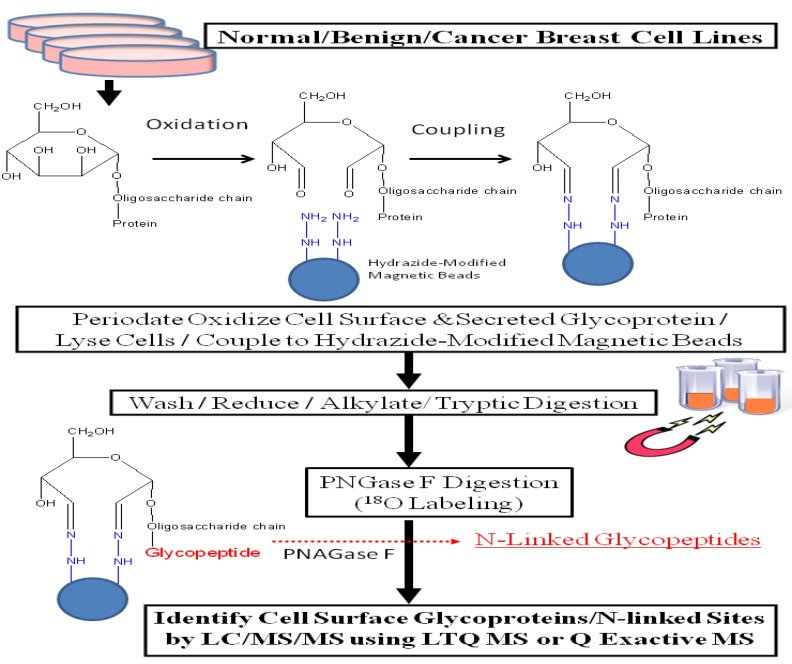
Experimental workflow scheme.

Compared to other proteins such as histones or cytosolic proteins, cell surface glycoproteins are present in low abundance in cells. Thus, an enrichment strategy is required in order to conduct in-depth glycoproteomic surveys via mass spectrometry. The authors previously demonstrated that incorporating periodate oxidation of intact cells, coupled with hydrazide-mediated enrichment [17] efficiently enriches for cell surface membrane and secreted glycoproteins from cell lines [18,19]. We optimized our protocol by incorporating hydrazide-modified magnetic beads [20,21] and used this revised protocol, illustrated in Figure 1, to analyze glycoprotein profiles derived from a normal human mammary epithelial population, two benign breast lines, and eleven breast cancer cell lines. This approach increased the identification of secreted and cell surface glycoproteins by more than 5-fold compared with results obtained through total cell lysate; glycoproteins constituted 57% *vs.* 11%, respectively, of all proteins identified through these two processes. These results were obtained with a linear ion trap-instrument (LTQ) featuring collision-induced dissociation (CID) to obtain tandem mass spectrometric analyses (CID-MS/MS). 

Recent advances in the design of mass spectrometers, coupled with significant improvements in mass accuracy, have substantially enhanced researchers’ ability to detect low abundant proteins in complex biological samples. To quantify the extent of this improvement, our first objective in the present study was to evaluate the Q Exactive MS, a new instrument which combines a quadrupole for selection of precursor ions for higher-energy collision-induced dissociation (HCD) with an Orbitrap for ion detection [22], for glycoprotein profiling.

### 2.1. The Q Exactive MS Delivers 2–3 Times as Many Cell Surface and Secreted Glycoprotein Identifications as the LTQ MS

**Figure 2:**
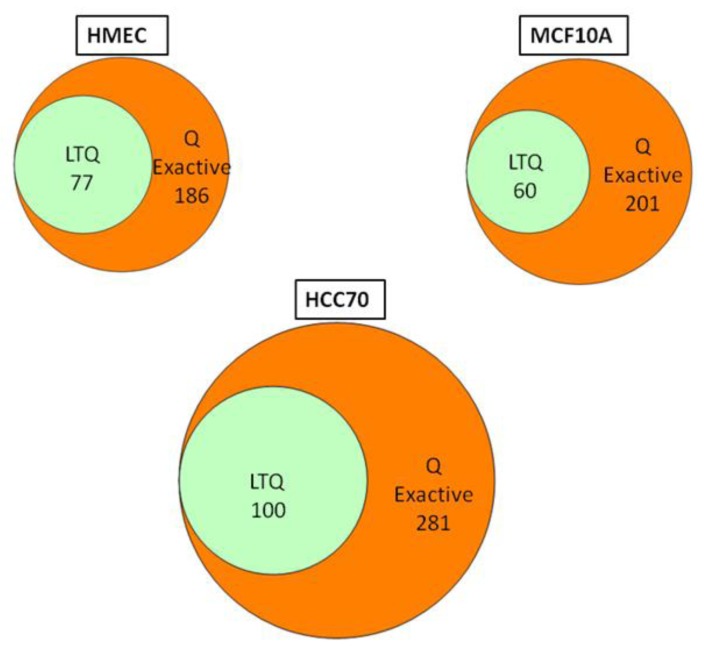
Venn diagrams of the glycoproteins identified by the Q Exactive MS and the linear ion trap-instrument (LTQ) MS from three breast cell lines. All glycoproteins found in LTQ MS analyses were also found in Q Exactive MS analyses.

We conducted glycoprotein profiling of the cell surface and secreted glycoproteomes using PNGase F-released glycopeptides from three cell lines: a normal mammary epithelial cell line (HMEC), a benign mammary epithelial line (MCF10A), and a breast tumor line (HCC70). We then analyzed identical samples from these three cell lines using single LC/MS/MS on the LTQ MS and on the Q Exactive MS for purposes of comparison. As shown in Figure 2, we identified an average of approximately 80 glycoproteins in our analyses of the samples run on the LTQ; in contrast, from the samples run on the Q Exactive, we identified 200–300 glycoprotein, a ~3-fold increase. Significantly, all 80 of the glycoproteins identified with the LTQ were also contained in the larger set identified with the Q Exactive. 

The Q Exactive has greater sensitivity for the detection of ions. This can be attributed to the improved design of its ion transmission lens and to its faster electronics which increase the number of MS/MS scans obtained during data acquisition. Q Exactive’s Orbitrap technology also provides more accuracy and higher resolution in mass measurement of precursors and their fragments than the LTQ. These technological enhancements are reflected in the detection of significantly greater numbers of glycopeptides using the Q Exactive*.*

The release by PNGase F of glycopeptides linked to the hydrazide resin catalyzes a deamidation reaction that results in the conversion of glycosylated Asn residues to Asp residues. Consequently, the total number of deamidated glycopeptides identified in our samples significantly exceeds the total number of identified glycoproteins because many of the glycoproteins contain more than one *N*-linked site (supplemental Table 1). HCD-MS/MS analyses of the HMEC, MCF10A, and HCC70 samples on the Q Exactive resulted in the identification of 827, 818, and 1,066 unique deamidated peptides, respectively, compared with 325, 153, and 292 identified via CID-MS/MS analysis on the LTQ. Glycoproteins detected with the Q Exactive were typically characterized by an average of 4 identified deamidated glycopeptides, compared with only 3 for the LTQ. This superior ability of the Q Exacative to identify deamidated peptides thus increases the reliability of its glycoprotein identifications.

**Figure 3:**
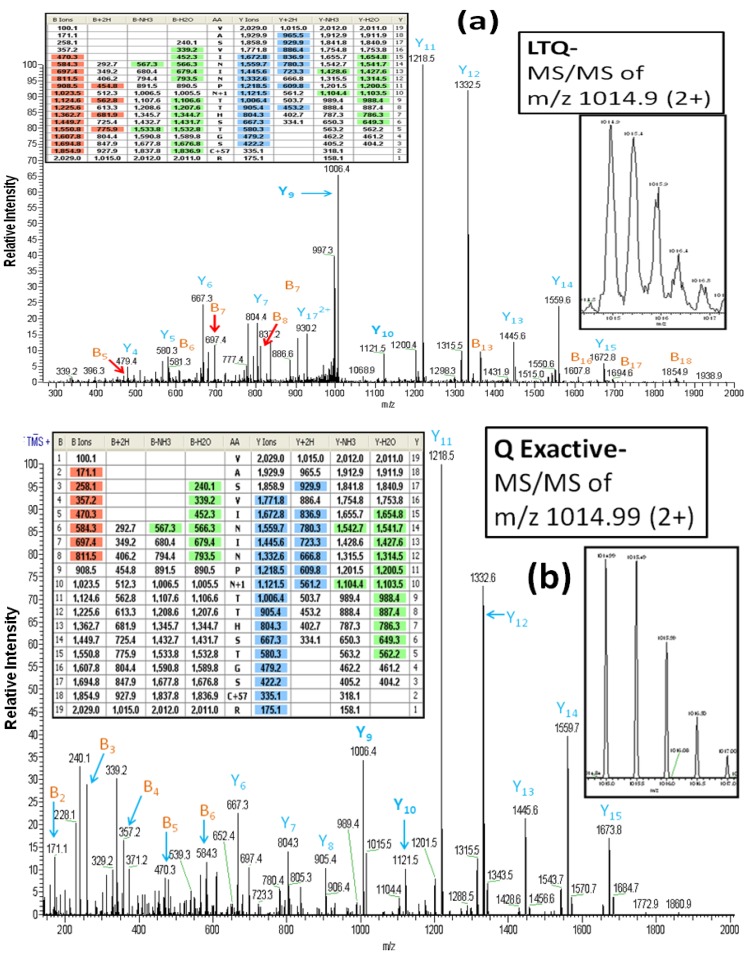
The LTQ MS CID-MS/MS spectrum (**a**) and the Q Exactive MS HCD-MS/MS spectrum (**b**) of the glycopeptide VASVININPN*TTHSTGSCR (AA#248-266, N* denotes a conversion of Asn to Asp for the *N*-linked site Asn^257^) from lysosome-associated membrane glycoprotein 2. Both spectra contain a rich set of structural-fragments. The inset figures show the isotopic profile of the doubly charged precursor ion, and the inset tables show lists of the color-coded fragments matching the expected values for various ions. B_n_ and Y_n_ denote the *N*-terminal and the B-terminal fragments, respectively. The MS/MS fragment nomenclature used is according to Johnson *et al.* [23].

As reported above, all of the glycoproteins identified in the LTQ analyses were also identified in the Q Exactive analyses. However, it is important to note that the MS/MS spectra which are the basis for the glycoprotein identifications differ between the two instruments. For example, Figure 3A (the inset figures show the precursor ion with its isotopic ions) depicts the CID-MS/MS spectrum obtained using the LTQ MS for the doubly charged ion of the glycopeptide (AA#248-266) from lysosome-associated membrane glycoproteins 2. This spectrum contains a rich set of structural-fragments similar to that generated in the HCD-MS/MS spectrum from the Q Exactive (Figure 3B). Most of y-series fragments are detected as relatively high abundance ions for both CID-MS/MS (y3-y15) and HCD-MS/MS (y1-y16) compared to the observed b-series fragments. However, more b-series fragments are observed in CID-MS/MS (b5-b18: See the color-coded B ions listed in the inset table in Figure 3A) compared to the HCD-MS/MS (b2-b8: See the color-coded B ions listed in the inset table in Figure 3B), whereas the HCD-MS/MS provides additional information (b2-b4) that is absent from the CID-MS/MS due to the low mass cutoff associated with the latter technology [24]. We find evidence of the conversion of Asn^257^ (NTT) to Asp by PNGase F in both the CID-MS/MS and HCCD-MS/MS spectra, as indicated by the mass difference of 115 Da between y9 and y10. The spectra also clearly show that two other Asn residues (Asn^253^ and Asn^255^) in the glycopeptide are not converted to Asp, in that a mass difference of 114 Da is present between y11 and y12, and between y13 and y14.

**Figure 4:**
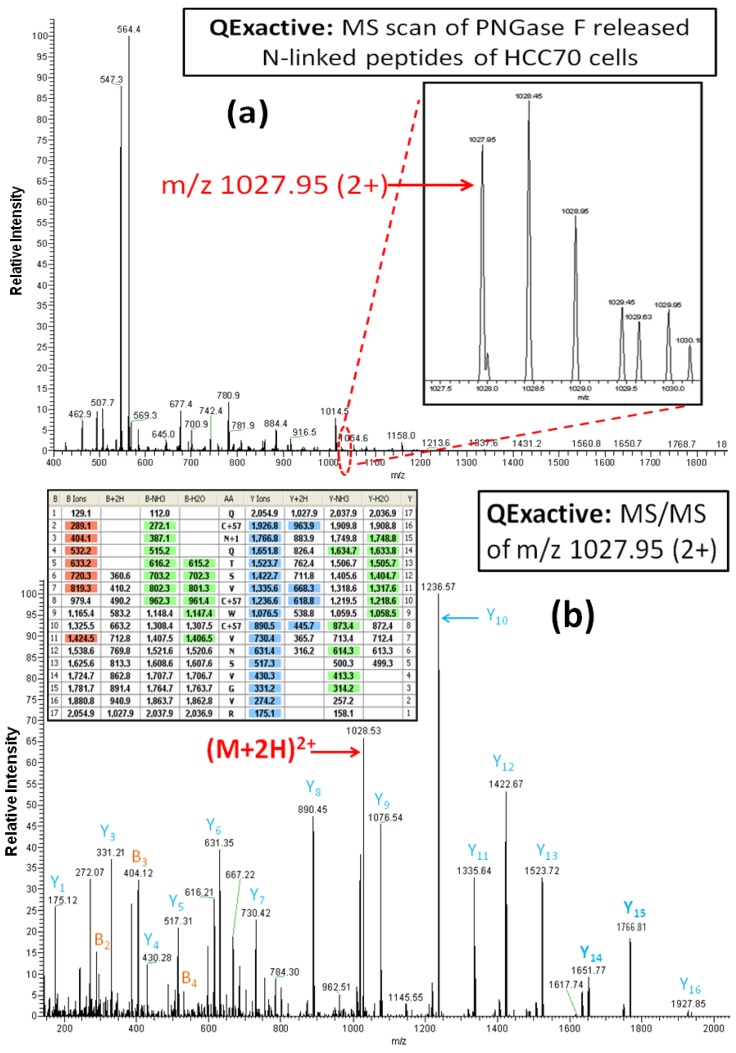
The Q Exactive MS spectrum (**a**) and the HCD-MS/MS spectrum (**b**) of the glycopeptide QCN*QTSVCWCVNSVGVR (AA#118-134, N* denotes a conversion of N to D for the *N*-linked site Asn^12^^0^) from tumor-associated calcium signal transducer 2. The mass difference of 115 Da between y14 and y15 confirms the conversion Asn^12^° to Asp by PNGase F.

Figure 4A,B illustrate one of the reasons for the significantly greater number of glycoprotein identifications by the Q Exactive MS than the LTQ MS. The *N*-linked glycopeptide containing amino acid residues of 118–134 (AA#118-134) from the tumor-associated calcium signal transducer 2 was detected in the sample from the breast cancer cell line HCC70 by the Q Exactive MS, but not by the LTQ MS. From the inset spectrum (Figure 4A) it is clear that this glycopeptide was identified from a relatively weak ion, representing less than 2% of the maximum ion intensity in the range of m/z 1027–1030.5. Although the intensity of the ion for this glycopeptide is weak, the isotopic pattern of the precursor ion, selected by data-dependent acquisition, is well resolved for a doubly charged ion with the monoisotopic peak occurring at m/z 1027.95. The HCD-MS/MS spectrum of the precursor ion at m/z 1027.95, shown in Figure 4B, reveals a complete set of y-series fragments (y1-y16). This peptide (AA#118-134) contains two Asn residues within its sequence. Based on the mass differences between y5 (517.31) and y6 (631.35) and between y14 (1651.77) and y15 (1766.81), we conclude that the Asn is converted to Asp, given the mass difference of 115.04 at Asn^12^^0^ (NQT), the *N*-linked consensus site. These results demonstrate the capacity of the Q Exactive MS to identify *N*-linked glycopeptides occurring in samples at very low abundance without ambiguity.

**Figure 5:**
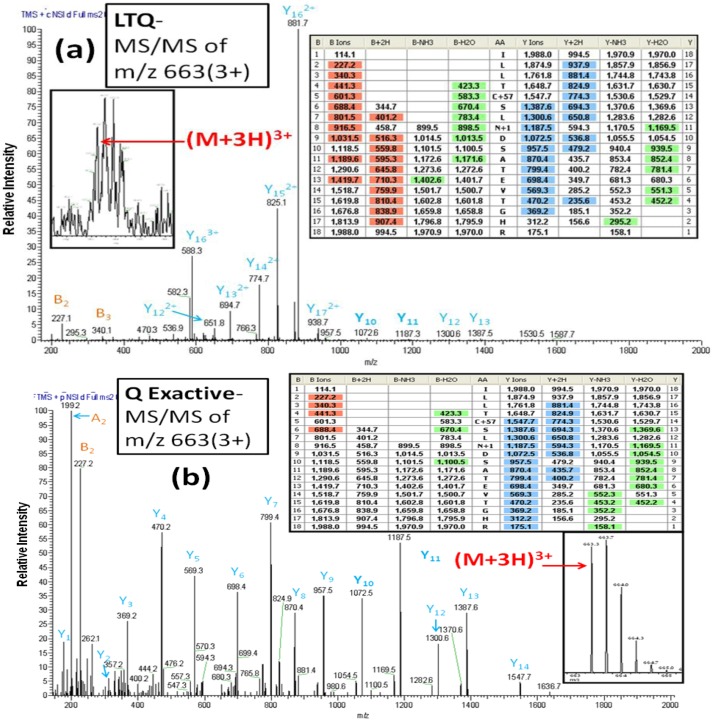
The CID-MS/MS spectrum (**a**) and HCD-MS/MS spectrum (**b**) of a triply charged precursor ion of the glycopeptide ILLTCSLN*DSATETGHR (AA#153-170, N* denotes a conversion of N to D for the *N*-linked site Asn^160^) from basigin produced by the LTQ MS and the Q Exactive MS, respectively. The CID-MS/MS spectrum (**a**) contains doubly charged y-series fragments and yields little information for an unambiguous identification of the glycosylation site.

In contrast to the LTQ MS, the Q Exactive MS offers higher resolution and more accurate mass measurements. The usefulness of these features is evident in the spectra obtained for a glycopeptide (AA#153-170) from the glycoprotein basigin or CD147. Figure 5A shows the CID-MS/MS for this *N*-linked peptide from the PNGase F-released fraction from the benign breast cancer cell line MCF10A. The inset zoom scan spectrum in Figure 5A shows the monoisotopic peak at m/z 663.3 of a triply charged ion for the basigin *N*-linked peptide (AA#153-170). A characteristic of CID-MS/MS spectra generated from the triply charged ions is the presence of an abundance of doubly *vs.* singly charged fragments. This is clearly shown by the dominant fragments ions y15 and y16 in Figure 5A. However, the identity of these multiply charged ions is difficult to determine with the LTQ MS because of its low resolution. In addition, the singly charged ions y11 and y10, which represent the conversion of Asn to Asp for the *N*-linked site Asn^160^ are barely detectable in the LTQ MS-generated spectrum. In contrast, the HCD-MS/MS fragments of the identical triply charged ion in the spectrum generated by the Q Exactive MS (Figure 5B), produce more singly charged, y-series fragments and also yield the information required for unambiguous identification of the glycosylation site, namely, the mass difference of 115 Da between y10 and y11. The inset spectrum in Figure 5B of the region of the spectrum between m/z 663 and 665 shows an enlargement of the full scan MS spectrum from m/z 400–1900 produced by the Orbitrap, demonstrating a superior mass resolution with mass accuracy of <5 ppm with the Q Exactive.

In summary, the results generated from the Q Exactive analyses (Figure 3, Figure 4, Figure 5) demonstrate that the detection limits of this newly developed instrument are dramatically superior to those of the LTQ ion trap. Two major factors contribute to this enhanced detection limit: the ability to detect low abundant ions; and the generation of more structural fragments by HCD for tryptic peptides containing singly charged state ions. It is clear from the results presented here that the Q Exactive MS significantly increases the depth of coverage of the cell surface and secreted glycoproteomes examined over the coverage generated by the LTQ ion trap MS. 

### 2.2. How Extensive Is the Misidentification of N*-*Linked Sites due to Chemical Deamidation?

PNGase F treatment of glycoproteins has been used to facilitate the identification of *N*-linked glycosylation sites and has resulted in a large database of *N*-linked glycosites [20,25,26]. This approach relies on the conversion of Asn to Asp residues within the consensus sequence NXT/S. Since deamidation of Asn residues can occur through a non-enzymatic process [12,15], false identifications can occur, particularly from high throughput analyses [14]. Recently, Palmisano *et al*. [13] have highlighted the potential for false positive *N*-linked glycosite identifications in their analysis of membrane proteins isolated from bacteria lacking the enzymatic machinery necessary for *N*-linked glycosylation. They concluded that greater caution should be exercised regarding the assignment of *N*-linked glycosylation sites in high throughput glycoproteomic studies. 

In response to this concern, we systematically investigated the false positive rate of *N*-linked glycosylation site identifications in the Q Exactive and the LTQ MS analyses discussed above. In contrast to the study by Palmisano *et al*. [13], our analyses—like most glycoproteomic analyses—incorporate a glycoprotein enrichment step into the sample preparation protocol. A common approach is to use periodate oxidation of the glycans attached to glycoproteins and to capture of the glycoproteins on a hydrazide resin (as shown in Figure 1 of the Experimental scheme). *N*-linked glycopeptides are released from the hydrazide resin using PNGase F following tryptic digestion of the coupled glycoproteins. 

At least three factors contribute to the false positive identification of *N*-linked glycosites: (1) the poor quality of MS or MS/MS spectra (weak signal, and/or interfering ions); (2) incorrect monoisotopic peak assignment for the precursor ion; and (3) the use of suboptimal searching parameters (precursor and product ions mass tolerance) in protein identification software. The first factor relates to the low abundance of *N*-linked glycopeptides available for MS analyses and results in an incorrect precursor ion peak assignment and/or a lack of MS/MS fragments for identifying true Asn to Asp conversion sites. The effect of this factor is reduced when the concentration of glycopeptides available for analyses is significant; thus, this is less of a concern when glycopeptide enrichment is used. 

The other two major factors contributing to the false positive identification of *N*-linked glycosites are the use of low resolution MS and MS/MS scans (LTQ MS in the present case) and the use of non-optimized searching parameters to identify deamidated Asn residues. To illustrate the effects of these two factors on the false positive identification rate of *N*-linked glycosite, we use here the results we obtained with the MCF10A breast cell sample. The 20 most abundant glycoproteins identified from this sample produced 487 MS/MS spectra detected with the Q Exactive MS, and 261 MS/MS spectra detected with the LTQ MS. Deamidated *N*-linked peptides were identified using the Mascot algorithm. We manually inspected each spectrum to determine whether the assignment of an Asn to Asp conversion was correct, and then used the results of this inspection to determine the rate of false positive assignments. The results are summarized in Table 1. 

**Table 1:** False positive identifications of deamidated sites using various Mascot searching parameters.

MS System	Mascot Precursor Ion Mass Tolerance	Mascot Product Ion Mass Tolerance	Average Mascot Ion Score	Average Mascot Identity Score	False Positive Rate(False Positive Spectra/Total Spectra)
LTQ	1.6 Da	0.8 Da	(2^+^ charge) 74.7	(2^+^ charge) 37.9	7.6% (18/238)
			(3^+^ charge) 51.2	(3^+^ charge) 37.7	15.4% (2/13)
Q Exactive	20 ppm	0.8 Da	(2^+^ charge) 69.2	(2^+^ charge) 29.9	3.4% (9/262)
			(3^+^ charge) 60.5	(3^+^ charge) 31.1	4.0% (9/225)
Q Exactive	20 ppm	0.05 Da	(2^+^ charge) 68.9	(2^+^ charge) 28.4	3.2% (8/252)
			(3^+^ charge) 58.7	(3^+^ charge) 29.7	3.3% (7/209)
Q Exactive	20 ppm	0.02 Da	(2^+^ charge) 66.8	(2^+^ charge) 28.4	1.4% (3/212)
			(3^+^ charge) 56.4	(3^+^ charge) 29.8	2.2% (4/185)
Q Exactive	20 ppm	0.01 Da	(2^+^ charge) 58.7	(2^+^ charge) 28.4	0.7% (1/147)
			(3^+^ charge) 52.2	(3^+^ charge) 29.8	0.8% (1/127)

We set Mascot searching parameters for the precursor and the product ion mass tolerance for MS/MS spectra generated from the LTQ at 1.6 and 0.8 Da, respectively. For the Q Exactive-generated spectra, we set the precursor ion mass tolerance at 20 ppm, and the product ion mass tolerance to vary between 0.8 Da and 0.01 Da (see Table 1). Varying the latter setting significantly affected the false positive rate of glycosite identification. The precursor ion mass tolerance for the LTQ MS is commonly set at 1.6 Da to maximize protein identifications. However, this tolerance value is greater than the mass difference resulting from Asn deamidation and results in a significant rate of misidentification (>7 and 15% for doubly and triply charged ions, respectively) of *N*-linked glycosites. The alternative of setting a narrower mass tolerance window reduces the number of false positive identifications, but will likely lead to the identification of fewer proteins (*i.e.*, an increase of false negatives). 

The mass accuracy for the full scan MS and the MS/MS spectra of the Q Exactive MS are less than 20 ppm and 0.1 Da, respectively. As shown in Table 1, the more accurate mass measurements obtained from the Q Exactive result in a substantially lower rate of false positives than those obtained from the LTQ. Reducing the product ion mass tolerance from 0.8 to 0.05 Da produces fewer false positive identifications and a more significant reduction in false positives are observed when the product ion mass tolerance is set at 0.01 Da. However, the number of MS/MS spectra for deamidated peptides is reduced by ~45% (274 *vs.* 487) at the lowest mass tolerance setting. These data clearly demonstrate that, with a high resolution instrument, the optimization of mass tolerance settings significantly reduces the rate of *N*-linked glycosite false positive identifications. 

**Figure 6:**
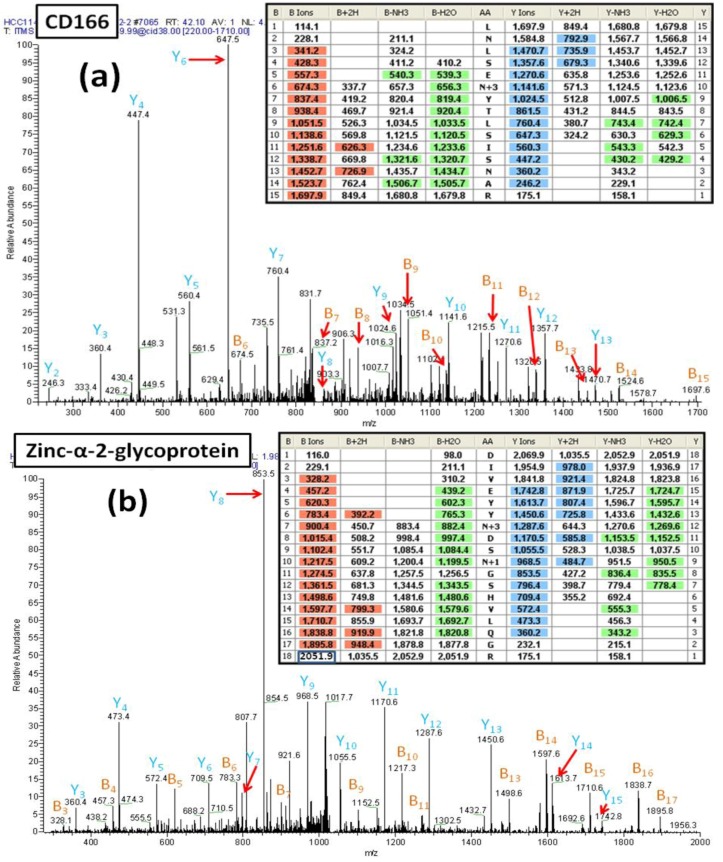
(**a**) The MS/MS spectrum of the CD166 glycopeptide LNLSEN^#^YTLSISNAR (AA#90-144, N^#^ denotes a conversion of N to D with ^18^O for the *N*-linked site Asn^95^) containing two *N*-linked consensus sequons. The detected mass difference of 117 Da between y9 and y10 demonstrates that Asn^95^ is deamidated, labeled with ^18^O and thus, was glycosylated. (**b**) The MS/MS spectrum of the zinc-α-2-glycoprotein glycopeptide DIVEYYN^#^DSN *GSHVLQGR (AA#103-120, N^#^ denotes a conversion of N to D with ^18^O for the *N*-linked site Asn^109^, and N* denotes a conversion of N to D for the *N*-linked site Asn^112^) that contains two *N*-linked consensus sequons. The detected mass difference of 117 Da between y11 and y12 demonstrates that the Asn^109^ is deamidated and is labeled with ^18^O thus is glycosylated. The detected mass difference of 115 Da between y8 and y9 demonstrates that the Asn^112^ is not glycosylated, and has undergone non-enzymatic deamidation.

To reduce the misidentification of *N*-linked glycosylation sites associated with the use of a low resolution instrument such as the LTQ, ^18^O water can be included in the PNGase F step. This results in a mass shift of ~3 Da when an Asn is converted to Asp. This approach is also useful for identifying *N*-linked glycosites in peptides with more than one *N*-linked consensus sequence. The latter application is illustrated in Figure 6A, Figure 6B.

We carried out PNGase F treatment in the presence of ^18^O water to release peptides from the hydrazide magnetic beads obtained from the HCC1143 sample. Figure 6A shows the MS/MS spectrum of a peptide (AA#90-104) from the CD166 glycoprotein that contains two *N*-linked consensus sequences and an additional Asn residue. The mass difference of 114 Da for fragments y2 and y3 demonstrates that the Asn^102^ is not deamidated. Observed masses for the fragments b5 and b6 show that ^91^Asn is not deamidated and thus is not a glycosylated site. In contrast, the mass difference of 117 Da between the fragment ions y9 and y10 (reflecting the mass of Asp labeled with ^18^O (115+2)) is consistent with deamidation of Asn^95^ and the incorporation of ^18^O. This spectrum provides clear evidence that the peptide contains one *N*-linked consensus site at Asn^95^ that is glycosylated. 

Figure 6B presents another example of how the incorporation of an ^18^O label can facilitate the correct identification of an *N*-linked glycosylation site within a peptide that contains multiple *N*-linked consensus sequences. This figure shows the MS/MS spectrum of a glycopeptide (AA#103-120) from zinc-α-2-glycoprotein. In this case, the results establish that one of two Asn residues is glycosylated, whereas the other is not glycosylated but has been converted to Asp via non-enzymatic deamidation. The detected mass difference of 117 Da between y11 and y12 shows that Asn^109^ is glycosylated, containing an Asp residue with ^18^O. In contrast, the mass difference of 115 Da from y8 to y9 is consistent with the deamidation of Asn^112^ to Asp, consistent with the non-enzymatic deamidation of Asn^112^. 

The results in Table 1 and Figure 6A,B demonstrate that utilizing a high resolution MS such as the Q Exactive, and incorporating ^18^O water into the PNGase F reaction mixtures, reduce the potential for misidentification of *N*-linked glycosylation sites within glycoproteins. Our results demonstrate that with a high resolution instrument and optimized mass tolerance settings, the false positive identifications of *N*-linked glycosylation sites occurs at a level of ~1%. 

## 3. Experimental Section

*Cell Culture*- Two breast cancer cell lines (HCC1143 and HCC70), a benign breast tumor cell line (MCF10A), and a normal human mammary epithelial cell line (HMEC), were grown in 10 cm culture dishes with 10 mL of culture medium. HCC1143, HCC70 and MCF10A cells and their culture media were supplied by American Type Culture Collection (ATCC, Manassas, VA, USA)**.** HMEC cells and its culture medium were supplied by Cell Applications, Inc. (San Diego, CA, USA). Cells were grown to over 90% confluence at 37 °C with 5% CO_2_ prior to being treated with periodate oxidation. Each sample (12 mL of lysate) for analysis was prepared from 15 culture dishes (10 cm), yielding about 1 mg of total protein. The biological replicates varied for the cell lines used in the study from 2–4 as follows: HMEC and HCC70, 2 biological replicates; MCF10A, 4 biological replicates. At least 5 technical replicates were used for each biological replicate.

*Periodate Oxidation*- Intact cells on culture dishes were treated with periodate to oxidize monosaccharides within the carbohydrate chains of secreted and cell surface glycoproteins as previously described. The growth medium was aspirated from each plate of cells, and the cells were rinsed once with 5 mL of phosphate buffered saline (PBS). Carbohydrates were oxidized with 3 mL of 10 mM NaIO_4_ (Sigma-Aldrich, St. Louis, MO, USA) in oxidation buffer (20 mM sodium acetate, 150 mM NaCl, pH 5) in the dark at 25 °C for 1 h with gentle rocking, and the periodate solution removed by aspiration. The cells were washed with 4 mL of PBS with gentle rocking for 2 min and the PBS buffer removed.

*Cell Lysate Preparation*- Periodate treated cells were incubated with 0.5 mL of lysis buffer containing a 1% octyl-β-D-1-thioglucopyranoside, 1% protease inhibitor cocktail (Sigma-Aldrich), 20 mM sodium acetate, and 150 mM NaCl at pH 5 at room temperature for 1 h with gentle rocking. Cell residue was scraped from the dishes and homogenized by multiple passes through a syringe with a series of different needle sizes ranging from 19 to 27½ gauges. The lysates were clarified by centrifugation at 14,000 rpm for 8 minutes at 4 °C, the supernatant was carefully collected, total protein content was determined using the Bradford assay, and the supernatant frozen at −20 °C until samples were enriched for glycoproteins using hydrazide magnetic beads. 

*Glycoprotein Enrichment using Hydrazide Magnetic Beads*-Breast cell lysates (12 mL) were spiked with 5 µg of periodate oxidized chicken ovalbumin (Sigma-Aldrich), which served as an internal control to monitor the coupling process. Hydrazide magnetic beads (30 mg, 1 µm size, Bioclone, San Diego, CA, USA) were diluted to 1ml with coupling buffer (20 mM sodium acetate, 150 mM NaCl, pH 5 adjusted using acetic acid) in a 2 mL centrifuge tube. The suspension was vortexed and the beads were recovered with a magnetic separator. The washing step was repeated, the beads were equally divided into eight 2 mL tubes, and each tube was mixed with 1.5 mL lysate. The suspension in the tubes was shaken for 16 h at 25 °C with a thermo mixer at 800 rpm. 

*Glycoprotein Reduction, Alkylation, Denaturation and Trypsin Digestion*-The magnetic beads were washed (1 mL per wash) with a series of three washing buffers (1.5 M NaCl, 60% methanol, 60% acetonitrile). Proteins bound to the magnetic beads were denatured with 8 M urea at 25 °C with constant mixing for 15 min. The bead washing and protein denaturalization was repeated once. The bound proteins were reduced with 1 mL of 35 mM dithiothreitol for 40 min at 40 °C. The beads were rinsed with 1 mL of 50 mM ammonium bicarbonate buffer and the reduced glycoproteins were alkylated with 1 mL of 50 mM iodoacetamide for 30 min at 25 °C in the dark with constant mixing. The iodoacetamide was discarded, and the immobilized glycoproteins rinsed with 1ml of ammonium bicarbonate buffer. The glycoproteins were digested with 0.5 mL of trypsin (20 ng/µL; Promega, Madison, WI, USA) in 50 mM ammonium bicarbonate buffer at 37 °C with constant mixing for 12 h. After digestion, the tryptic fraction was collected, and the hydrazide magnetic beads were washed with 3 mL of 50 mM ammonium bicarbonate to collect any remaining tryptic peptides. 

*N*-*Glycopeptide Release from the Hydrazide Resin with N*-*Glycosidase F*-The *N*-linked glycopeptides bound to the hydrazide magnetic beads were released from the beads with 4 µL *N*-glycosidase F by incubating the solution overnight at 37 °C with constant mixing. PNGase F was supplied by Prozyme (Glyco *N*-Glycanase 200mU, Hayward, CA, USA) and was dilute 4x with 50 mM ammonium bicarbonate buffer before use. The released *N*-linked glycopeptide fraction was collected and the beads were rinsed with 3 mL of 50 mM ammonium bicarbonate buffer. The ammonium bicarbonate rinse solution was collected and combined with the PNGase F released fraction, and subsequently processed by solid phase extraction (SPE) to re-concentrate the sample. For the ^18^O labeling experiment, the *N*-linked glycopeptides bound to the hydrazide magnetic beads were heated to 95 °C for 5 min and dried by speedvac, followed by the addition of H_2_^18^O (500 µL) and PNGase F (4 µL). The conditions for the PNGase F treatment with H_2_^18^O were the same as described above. After digestion the solution was collected, and processed by SPE. 

*Solid Phase Extraction*-The *N*-linked glycopeptide fractions were each subjected to SPE (Strata-X reversed phase, Phenomenex, Torrance, CA, USA). The SPE resin was activated with 1ml of HPLC grade methanol and rinsed with 1 mL of HPLC grade water to remove the remaining methanol. The PNGase F treated *N*-linked glycopeptides, were loaded onto and bound to an SPE column using a flow rate of 1–2 mL/min. The resin-bound peptides were rinsed with 2 mL of HPLC grade water to remove salts, eluted with 1ml of 65% methanol/water, the eluent dried using a Speed-Vac apparatus, and stored at 4 °C prior to mass spectrometric analysis.

*Peptide/Protein Identifications by ESI*-*MS/MS Analysis*- The deglycosylated *N*-linked peptides derived from each cell lysate were separately analyzed by liquid chromatography/electrospray ionization-tandem mass spectrometry (LC/ESI-MS/MS). The dried samples of the PNGase F released *N*-linked glycopeptides were dissolved with 50 µL of 0.1% formic acid/water. 5 µL of each sample was analyzed by LC/ESI-MS/MS using a Thermo LTQ ion trap (Thermo Fisher, San Jose, CA, USA) mass spectrometer with a dual Thermo Surveyor HPLC pump system or a Q Exactive (Thermo Fisher) mass spectrometer with a NanoLC system using data dependent acquisition with dynamic exclusion settings of DE = 1, 30 s for LTQ and 10 s for Q Exactive, respectively. The optimization for selecting dynamic exclusion is based on Zhang *et al.* [27] The data dependent acquisition settings used were a triple play-top 4 CID for the LTQ MS, and a top12 higher energy collision induced dissociation (HCD) for the Q Exactive MS, respectively. Resolving power for LTQ with a zoom scan was ~5,000. Resolving power for Q Exactive was set as 70,000 for the full MS scan, and 17,500 for the MS/MS scan at m/z 200. LC/ESI-MS/MS analyses were conducted using a C18 column (75 μm × 130 mm). The mobile phases for the reverse phase chromatography were (A) 0.1% HCOOH/water and (B) 0.1% HCOOH in acetonitrile. A four-step, linear gradient was used for the LC separation (5% to 35% B in the first 65 min, followed by 35% to 80% B in the next 10 min, holding at 80% B for 5 min, and return to 5% B during the final 10 min). The Mascot (v2.3) [28] algorithm was used to identify peptides from the resulting MS/MS spectra by searching against the combined human protein database (a total of 22,673 proteins) extracted from SwissProt (v57.14; 2010 February) using taxonomy “homo sapiens” (22,670 proteins). BSA and fetuin provided a means for estimating the level of protein contamination resulting from fetal bovine serum proteins contained in the cell culture medium. Ovalbumin was used to estimate glycoprotein recovery. Searching parameters for parent and fragment ion tolerances were set as 1.6 and 0.8 Da for the LTQ MS, and 20 ppm, and varying values (see Table 1) between 0.01–0.8 Da for the Q Exactive MS. Other parameters used were a fixed modification of carbamidomethyl-Cys, variable modifications of deamidation-Asn, and oxidation-Met. Trypsin was set as the protease with a maximum of 2 missed cleavages. Scaffold (Proteome Software) was used to merge and summarize the data obtained from the LC/MS/MS protein identification analyses for the LTQ MS and the Q Exactive MS. Protein identifications were based on a minimum detection of 2 peptides with 99% protein identification probability using the algorithm ProteinProphet [29]. Each peptide identified had a minimum peptide identification probability of 95% using the algorithm PeptideProphet [30]. The average false positive rate for the peptide identification in this study was less than 5% for LTQ and less than 1% for Q Exactive based on results obtained with PeptideProphet. ProteinID Finder (Proteome Solutions) was used to determine whether the peptide was derived from a glycoprotein from the UniProt database for each identified protein. A summary of the experimental flow work is shown in Figure 1.

## 4. Conclusions

This paper presents results of the analysis of glycoproteins using the new Q Exactive MS. Our data demonstrate that this high resolution/accurate mass instrument provides deep glycoproteomic coverage from breast cell line lysates, and that such coverage extends far beyond that obtainable with the low-resolution LTQ MS. Specific advantages of the Q Exactive include enhanced instrument sensitivity resulting from improvements in the design of the ion transmission lens, and faster electronics which increase the number of MS/MS scans during data acquisition. We provide detailed data which show that the Q Exactive increases the detection of *N*-linked glycopeptides in samples at very low abundance without any structural assignment ambiguity, that optimization of its mass tolerance settings significantly reduces the misidentification of *N*-linked glycosites, and that incorporating ^18^O labeling into the PNGase F release of glycopeptides offers further advantages.

## Supplementary Files

Supplementary File 1 Supplementary Information (XLSX, 27 KB)
